# Herpes Zoster Following Chemo-Immunotherapy With Pembrolizumab in Metastatic Non-small Cell Lung Cancer: A Case Report

**DOI:** 10.7759/cureus.106695

**Published:** 2026-04-09

**Authors:** Andy Kouanga, Hamadoun Traoré, Choukri Elm'hadi, Hassan Errihani, Rachid Tanz

**Affiliations:** 1 Department of Medical Oncology, Mohammed V Military Teaching Hospital, Rabat, MAR; 2 Department of Medical Oncology, National Institute of Oncology, Rabat, MAR

**Keywords:** case report, herpes zoster, immune checkpoint inhibitors, lung neoplasms, pembrolizumab, varicella-zoster virus

## Abstract

Introduction: Chemo-immunotherapy has reshaped the treatment of metastatic non-small cell lung cancer (NSCLC), yet data on varicella-zoster virus (VZV) reactivation under these regimens are limited. Major immunotherapy trials do not specifically describe herpes zoster events in patients receiving combined chemotherapy and immune checkpoint inhibitors.

Methods: We describe a case of metastatic lung adenocarcinoma (ADC) treated with first‑line Pemetrexed-Carboplatin-Pembrolizumab plus Denosumab in which herpes zoster occurred during treatment, detailing clinical course, management, and outcomes.

Results: We report a 64-year-old former smoker with metastatic lung ADC involving lymph nodes and bone, Programmed Cell Death Protein Ligand (PD-L1) expression 0%, and no actionable oncogenic alterations. He was treated with first-line Pemetrexed-Carboplatin-Pembrolizumab plus Denosumab. Treatment was well tolerated, and post-cycle 4 imaging showed stable disease according to the Response Evaluation Criteria in Solid Tumor (RECIST) criteria. Approximately four months after initiation of chemo-immunotherapy, he developed a painful, unilateral, dermatomal vesicular eruption over the right lower thorax, clinically consistent with herpes zoster. The diagnosis of herpes zoster was clinical rather than microbiological. Systemic anticancer therapy was temporarily suspended. The patient received oral Acyclovir, step II analgesics, and local wound care for 14 days. Complete resolution of skin lesions and pain occurred within three weeks, without zoster-related complications. Maintenance Pemetrexed-Pembrolizumab-Denosumab was then resumed, with sustained tumor control and no recurrence of herpes zoster during follow-up.

Conclusion: This case illustrates herpes zoster reactivation during Pembrolizumab-based chemo-immunotherapy in metastatic NSCLC. It emphasizes the importance of systematically excluding infectious causes in new cutaneous eruptions under immunotherapy and supports multidisciplinary management. These findings also highlight the need to further explore VZV screening and vaccination strategies in cancer patients undergoing immunosuppressive treatments.

## Introduction

Immunotherapy has revolutionized the management and prognosis of cancers, particularly metastatic non-small cell lung cancer (NSCLC). Its combination with chemotherapy is now an integral component of the treatment of lung cancer without targetable oncogenic alterations. In KEYNOTE‑189, severe cutaneous adverse events were reported in approximately 2% of patients receiving Pembrolizumab with or without chemotherapy, although varicella-zoster virus (VZV) reactivation was not specifically described [1,2]. Immune checkpoint inhibitors (ICI) enhance the immune system by promoting the proliferation of effector T lymphocytes, while cytotoxic chemotherapy agents induce cell death. The blockade of inhibitory molecules PD-1 and CTLA-4, combined with chemotherapy, potentiates the cytotoxic response. However, these therapeutic effects are not devoid of adverse events [3,4].

The VZV can reactivate spontaneously or in response to various triggering factors, causing herpes zoster [5]. Cancer patients are often immunocompromised due to their disease or its treatment [6,7].

Some studies have described and examined viral zoster reactivation in patients with hematologic malignancies under treatment, such as lymphomas; others have also examined viral zoster reactivation with either immunotherapy alone or chemotherapy alone [6-8]. However, the literature does not specifically address VZV reactivation in patients with solid tumors receiving combined immunotherapy and chemotherapy.

We report a case of herpes zoster reactivation in a patient with metastatic lung adenocarcinoma (ADC) treated with a combination of chemotherapy and a PD-1 inhibitor in order to discuss the clinical management and implications.

## Case presentation

A 64-year-old married man, father of five children, with a chronic smoking history of 48 pack-years (quit three months before admission) and unknown vaccinal status, followed for benign prostatic hyperplasia under Tolterodine 4 mg for two years, with no history of tuberculosis exposure or family cancer history, was referred by the pneumology department of the Mohammed V Military Training Hospital for the management of metastatic lung ADC.

The patient’s history dated back to July 2023, marked by the onset of low-volume hemoptysis associated with exertional dyspnea evolving in the context of a declining general condition. Diagnostic workup included a chest computed tomography (CT) scan revealing a suspicious pulmonary process in the left lower lobe. Pulmonary biopsy with histopathological examination and immunohistochemical analysis confirmed a lung ADC.

18-Fluorodeoxyglucose (18-FDG) positron emission tomography (PET-CT) demonstrated hypermetabolic lesions consistent with a poorly defined parenchymal pathological process involving the left lower lobe (48 × 40 mm), associated with pathological lymphadenopathy in the right and left supraclavicular, mediastinal, and pre-tracheal nodal areas, as well as a hypermetabolic focus in the right acetabulum. Brain magnetic resonance imaging (MRI) was unremarkable.

On oncological referral, the patient was in good general condition (Eastern Cooperative Oncology Group, Performance Status ECOG PS 1), hemodynamically and respiratory stable (oxygen saturation 98% on room air), and complained of severe pelvic pain (Numerical Rating Scale 7/10). He received palliative radiotherapy to the right acetabulum combined with oral step-II analgesics. A complete laboratory workup was normal, including complete blood count, electrolytes, renal, thyroid, and hepatic function tests, as well as morning cortisol. HIV and hepatitis serologies (HBV, HCV, and HAV) were negative. PD-L1 expression was 0%, and no actionable oncogenic mutation was identified (epidermal growth factor receptor (EGFR) and anaplastic lymphoma kinase (ALK) negative).

The patient was therefore managed for stage IV lung ADC with lymph node and bone involvement, without targetable oncogenic alterations, and with PD-L1 expression at 0%.

He received four cycles of Pemetrexed 800 mg/21 days, Carboplatin 500 mg/21 days, Pembrolizumab 200 mg/21 days, and Denosumab 120 mg/21 days, initiated on November 1, 2023 (cycle 1), and completed on January 2, 2024 (cycle 4). Tolerance between cycles was good, and post-cycle 4 thoraco-abdomino-pelvic CT scan confirmed lesion stability per the Response Evaluation Criteria in Solid Tumor (RECIST) criteria.

**Figure 1:**
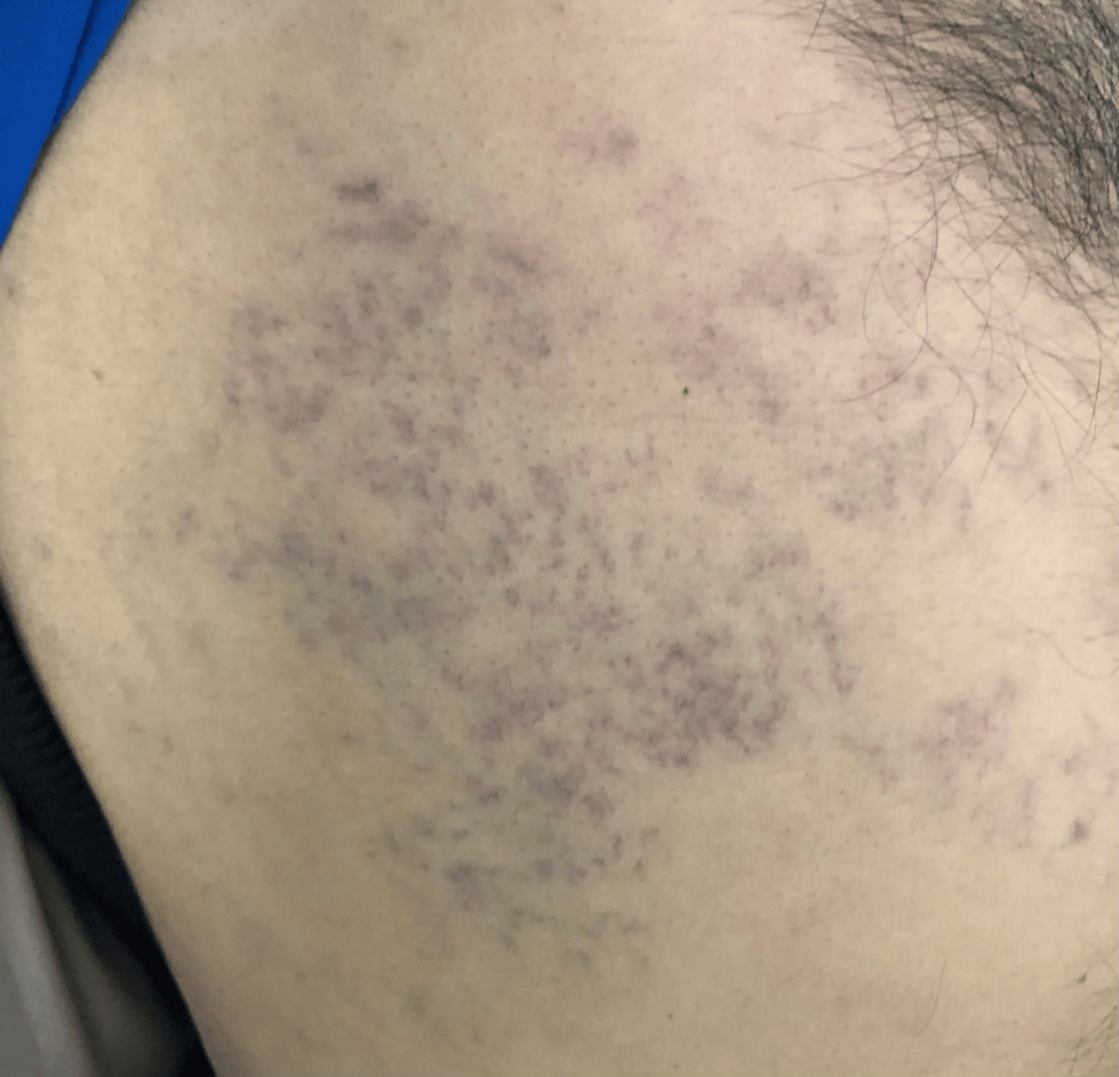
Skin lesions before herpes zoster treatment. Herpes zoster lesions before treatment, showing grouped vesicular eruptions on erythematous plaques in a right lower thoracic dermatomal distribution.

**Figure 2:**
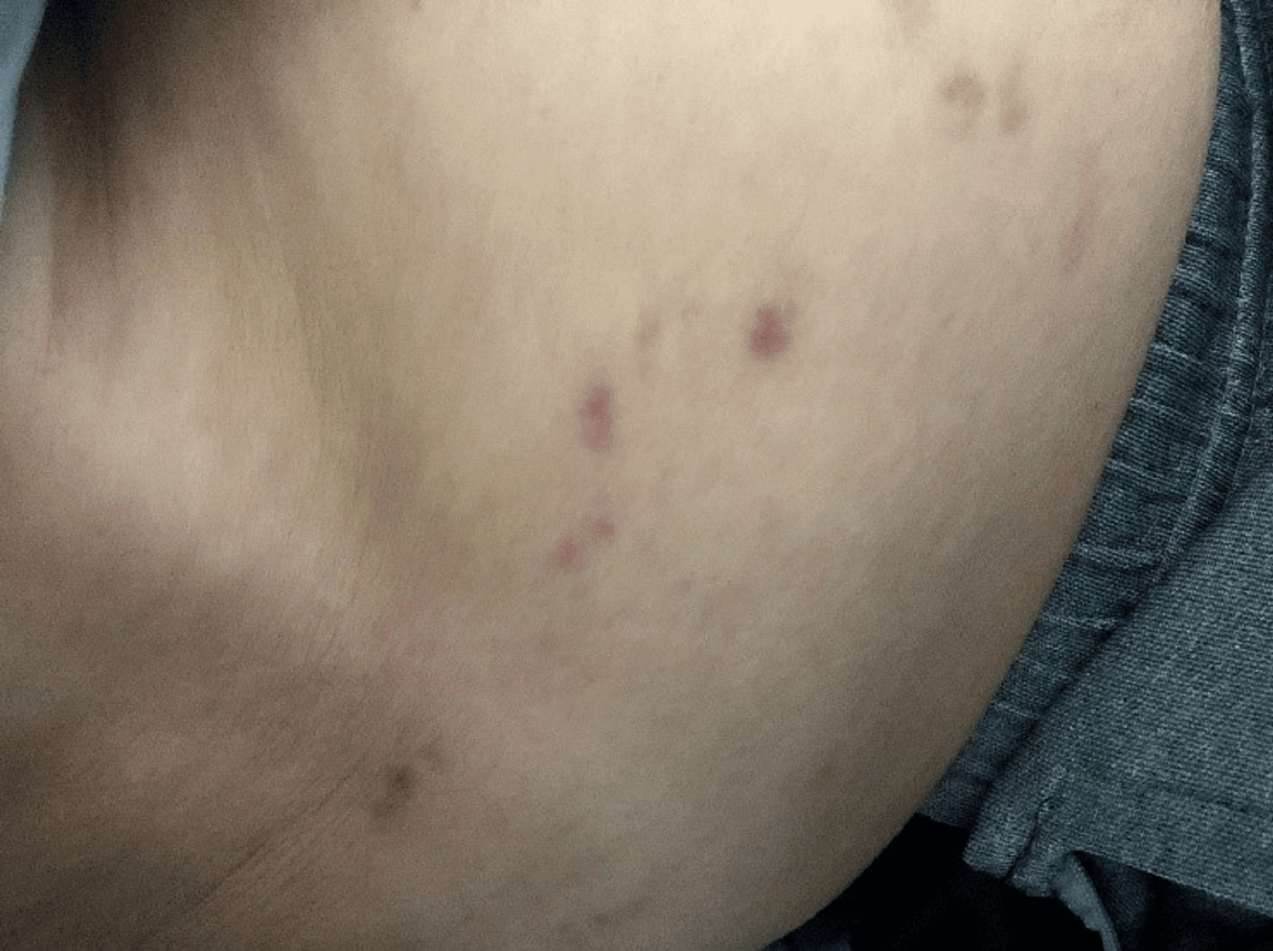
Skin lesions after herpes zoster treatment. Complete resolution of herpes zoster lesions three weeks after antiviral therapy, with residual post-inflammatory hyperpigmentation along the same dermatomal area.

The patient subsequently continued maintenance therapy with Pemetrexed, Pembrolizumab, and Denosumab. At cycle 5 of chemo-immunotherapy on February 13, 2024, clinical examination revealed grouped vesicular cutaneous eruptions on erythematous plaques, painful and band-like in a dermatomal distribution, unilateral over the right lower thorax (Figure 1). After specialist dermatological consultation, differential diagnoses (drug-induced toxicoderma, herpes simplex infection, and eczema) were excluded. Based on the typical unilateral, dermatomal, vesicular eruption and the presence of acute neuropathic pain, the diagnosis of herpes zoster was made clinically. CD4/CD8 lymphocyte subsets were not systematically monitored in this patient. Additional virological confirmation (PCR from skin lesions) was not pursued because the presentation was classic and management would not have been modified. Moreover, the patient was clinically stable, without systemic signs or atypical features that would mandate further diagnostic investigations. Anticancer treatment was suspended, and the patient was initiated on Acyclovir 800 mg five times daily for 14 days, step-II analgesics, and regular local wound care. Three weeks after treatment initiation, clinical improvement was marked by the complete disappearance of zosterian skin lesions (Figure 2) and the resolution of all previously described symptoms.

Maintenance therapy with Pemetrexed 800 mg/21 days, Pembrolizumab 200 mg/21 days, and Denosumab 120 mg/21 days was then resumed. The patient continued his treatment without further complications and with good disease control. The chronology of clinical events is summarized in Table 1.

**Table 1:** Chronology of clinical events. ADC: adenocarcinoma; RECIST: Response Evaluation Criteria in Solid Tumor.

Date/Time	Clinical Event
July 2023	Hemoptysis, exertional dyspnea, and a suspicious chest CT scan
Aug-Sep 2023	Biopsy: metastatic lung ADC
Nov 1, 2023	Start of Pemetrexed-Carboplatin-Pembrolizumab-Denosumab (cycle 1)
Jan 2, 2024	Cycle 4 completed; lesion stability (RECIST)
Feb 13, 2024	Cycle 5: right lower-thoracic zosterian eruption
Feb 2024	Treatment suspension; Acyclovir 800 mg × 5/day for 14 days
+3 weeks	Complete lesion resolution; maintenance therapy resumed (Pemetrexed-Pembrolizumab-Denosumab)
Follow-up	Treatment continued without herpes zoster recurrence; sustained tumor control

## Discussion

Our case involves a 64-year-old adult, consistent with a Japanese study by Taoka et al., conducted in lung cancer patients, which reported a mean age of herpes zoster onset of 62.5 years in patients receiving ICI and 73.7 years in those receiving chemotherapy alone. In that study, there was no significant difference in the incidence of herpes zoster between patients treated with immunotherapy alone versus chemotherapy alone (4.5% and 5%, respectively) [9,10]. Importantly, the Japanese series by Taoka et al. did not evaluate patients treated with combined chemo‑immunotherapy, thereby limiting direct extrapolation to regimens such as Pembrolizumab plus platinum‑pemetrexed.

In our patient, the time between treatment initiation and disease onset was approximately four months (November 2023 to February 2024), consistent with previous studies reporting a median time to herpes zoster onset after ICI administration of 135 days (4.5 months) and 121 days (4.1 months), respectively [11-13].

The association between cancer and herpes zoster is well established, largely explained by the reduction in immune efficacy resulting from the malignancy itself and its treatment. Large-scale studies have reported that cancer patients have a significantly increased risk of developing herpes zoster (up to 40%), and this risk is further elevated in patients receiving chemotherapy [6,7].

In prior population-based studies, factors associated with an increased risk of herpes zoster included advanced age, underlying disease, and drug-induced suppression of cellular immunity [8].

The pathophysiological explanation for VZV reactivation during immunotherapy is likely immune reconstitution inflammatory syndrome (IRIS), which induces an excessive immune response against infectious or non-infectious antigens by increasing T lymphocytes during a phase of immunosuppression. Thus, the clinical symptoms do not reflect active viral replication per se, but rather, the immune response directed against viral antigens [14]. In our case, the triggering factor was ICI-induced robust immune reconstitution.

Denosumab is primarily used for bone-targeted therapy; its effects on osteoclasts and the bone marrow microenvironment may contribute to mild immunomodulation, and a potential role in infection susceptibility cannot be entirely excluded in this context. In accordance with National Comprehensive Cancer Network (NCCN) and European Society for Medical Oncology (ESMO) guidelines, our patient received the KEYNOTE-189 regimen, whose pivotal trial demonstrated a significant overall survival (OS) benefit in favor of Pembrolizumab-chemotherapy combination (five-year OS rate of 19.4% vs. 11.3% in the intention-to-treat population) in treatment-naive patients with non-squamous NSCLC without EGFR/ALK mutations, regardless of PD-L1 score [2]. Patients with active infections were excluded from the trial, and severe cutaneous adverse events occurred in 2% of patients receiving immunotherapy with or without chemotherapy; however, infectious viral reactivations were not specifically reported [2].

The diagnosis of VZV infection is generally made clinically, except in atypical cases where PCR or skin lesion culture is used, based on the characteristic appearance of the eruption. Herpes zoster typically presents as a painful, unilateral, vesicular, dermatomal rash that resolves within a few weeks [5,15]. Treatment in accordance with the recommendations of dermatological societies was initiated. Standard antiviral therapy for herpes zoster consists of Acyclovir, while cidofovir or foscarnet should be used in cases of Acyclovir resistance. Anti-inflammatory and analgesic agents, including non-steroidal anti-inflammatory drugs, are used to manage acute pain [15].

Per American Society of Clinical Oncology (ASCO) clinical practice guidelines, infections and systemic diseases must be excluded in patients receiving immunotherapy who present with cutaneous reactions of any grade [11,13]. For optimal management, VZV and hepatitis virus screening in all cancer patients before initiating immunotherapy or other immunosuppressive treatments is recommended [8,15].

Herpes zoster is a vaccine-preventable disease; however, the role of vaccination in patients undergoing immunotherapy remains unclear. The clinical and economic consequences of herpes zoster in the context of increasing ICI use warrant further investigation, and oncologists should be sensitized to this issue. As immunocompromised patients generally experience more severe complications from herpes zoster, VZV vaccination is increasingly encouraged. The live attenuated zoster vaccine is contraindicated under immunosuppression, while the inactivated recombinant zoster vaccine is currently being evaluated in cancer patients undergoing active treatment [8,15].

The patient’s vaccination history against VZV was not documented in the medical record, which represents a limitation of this report.

## Conclusions

This case underscores the possibility of herpes zoster reactivation in patients treated with chemotherapy combined with a PD‑1 inhibitor and the need to systematically investigate infectious causes in any new cutaneous eruption occurring under immunotherapy. It highlights the importance of close multidisciplinary collaboration between oncologists, infectious disease specialists, and dermatologists for optimal evaluation and management. Finally, future prospective cohort studies should ideally incorporate baseline VZV serology and carefully timed administration of recombinant zoster vaccine to better define optimal prevention strategies in patients receiving chemo‑immunotherapy.
